# An advanced multimodal image fusion model for accurate detection of Alzheimer's disease using MRI and PET

**DOI:** 10.3389/fmedt.2025.1699821

**Published:** 2025-12-02

**Authors:** Arshiya S. Ansari, Mohammad Sajid Mohammadi, Carlo Cattani, Asifa Tassaddiq

**Affiliations:** 1 Department of Information Technology, College of Computer and Information Sciences, Majmaah University, Al Majmaah, Saudi Arabia; 2 Department of Computer Science, College of Engineering and Information Technology, Onaizah Colleges, Qassim, Saudi Arabia; 3 Department of Economics, Engineering, Society and Business Organization (DEIM), University of Tuscia, Viterbo, Italy; 4 Department of Mathematics and Informatics, Azerbaijan University, Baku, Azerbaijan; 5 Department of Computer Science, College of Computer and Information Sciences, Majmaah University, Al Majmaah, Saudi Arabia

**Keywords:** Alzheimer’s disease (AD), multimodal image fusion, magnetic resonance imaging (MRI), Glowworm Swarm-Optimized Spatial Multimodal Attention-Enriched Convolutional Neural Network (GWS-SMAtt-ECNN), positron emission tomography (PET)

## Abstract

The accurate detection of Alzheimer's disease (AD), a progressive and irreversible neurodegenerative disorder, remains a critical challenge in clinical neuroscience. The research aims to develop an advanced multimodal image fusion model for the accurate detection of AD using positron emission tomography (PET) and magnetic resonance imaging (MRI) techniques. The proposed method leverages structural MRI and functional 18-fluorodeoxyglucose PET (FDG-PET) information derived from the Alzheimer’s Disease Neuroimaging Initiative (ADNI). After preprocessing, including Gaussian filtering, skull stripping, and intensity normalization, voxel-based morphometry (VBM) is applied to extract gray matter (GM) features relevant to AD progression. A GM mask generated from MRI is used to isolate corresponding metabolic activity in the PET scans. These features are then integrated using a mask-coding strategy to construct a unified representation that captures both anatomical and functional characteristics. For classification, the model introduces a Glowworm Swarm-Optimized Spatial Multimodal Attention-Enriched Convolutional Neural Network (GWS-SMAtt-ECNN), where the optimization enhances both feature selection and network parameter tuning. The Python was implemented, and the result demonstrates that the proposed multimodal image fusion strategy outperforms traditional unimodal and basic fusion approaches in terms of F1-score (94.22%), recall (96.73%), and accuracy (98.70%). These results highlight the therapeutic usefulness of the suggested improved fusion architecture in facilitating immediate and accurate AD detection by MRI and PET.

## 1 Introduction

Alzheimer’s disease (AD) is the most prevalent cause of dementia, leading to 60%–80% of cases globally. It is a degenerative neurological illness. AD is characterized by memory loss, cognitive dysfunction, and changes in behavior, and it increases the strain on caregivers and healthcare systems and has a detrimental impact on patients' quality of life (QoL) (1). Early and accurate diagnosis of Alzheimer's is vital to apply effective interventions that can help improve the prognosis for the patient. Conventional diagnostic approaches to AD rely heavily on clinical interviews, memory tests, and behavioral observations (2). These assessments can be highly biased and can be unable to identify the disease at its earliest stages, when intervention can be most effective. By offering targeted, quantifiable, and non-invasive evaluations of brain structure and function, neuroimaging techniques such as MRI and positron emission tomography (PET) have completely changed the diagnosis of AD. MRI can visualize anatomical changes, such as hippocampal atrophy and cortical diminishing, as well as some functional changes (3). PET can show metabolic activity and markers of protein deposition (e.g., amyloid-beta and tau). These imaging techniques have the possibility to significantly increase the accuracy of diagnoses, enhance the assessment of disease progression, and help track how well treatments are functioning. MRI is a crucial imaging technique to appropriately diagnose and track symptoms in individuals (4). AD is studied using imaging techniques due to its capacity to generate fine-grained images of the brain's structure. The anatomical alterations correlated with AD, including ventricular enlargement, cortical thinning, and hippocampus atrophy, which impair memory and cognitive function, can be more effectively detected by MRI. Additionally, MRI modalities improve the utility for diagnosis, such as Diffusion Tensor Imaging (DTI), which assesses white matter (WM) integrity to show disruptions in neural connectivity, or by using functional MRI (fMRI) (5, 6). Techniques that measure brain activity through the use of tasks have shown functional deficits even in the absence of explicit structural deficits, supporting early and precise diagnosis. PET imaging, especially with radiotracers like FDG and amyloid-binding radiotracers, provides insight regarding the metabolism and molecular processes in the brain (7). FDG-PET shows areas of reduced glucose metabolism in regions of the brain that show pathology, while amyloid and tau PET imaging provide a visual representation of areas of brain pathologies with the accumulation of pathological proteins present in the brain (8). Early in the development of the disease, even before clinical symptoms appear, it can be visualized. The fusion of MRI and PET images produces a full image of structural and functional alterations in brain function. Multimodal imaging also enhances diagnostic accuracy for distinguishing AD from other dementias and provides developing therapy tailored to individual patients. With developments in approaches, integration of MRI and PET data allows for increasing success in automated and early detection of degenerative dementias such as AD (9, 10). The accurate early detection of AD remains very difficult and can remain a challenge due to the complexity of AD pathology, data heterogeneity, and limited treatment options. Conventional techniques are not accurate enough, and innovative multimodal techniques, utilizing MRI and PET imaging, DL, and multimodal feature fusion, are more accurate; there remain additional challenges related to robust, scalable, and comprehensible structures to increase the precision of illness advancement prediction and diagnosis. The goal of the research is to create a sophisticated multimodal image fusion model that integrates MRI and PET imaging to improve the specificity of AD detection. By gaining both structural and functional brain abnormalities, the model aims to enable more informative diagnosis and ultimately improve clinical decision-making through deep learning (DL)-powered analysis.

### 1.1 Key contribution

- An advanced image fusion model that integrates structural MRI and functional FDG-PET imaging for comprehensive AD analysis
- Applied voxel-based morphometry (VBM) to extract GM features from MRI, which are used to guide the fusion process and isolate consistent metabolic movement in PET images
- Considered a GWS-SMAtt-ECNN to enhance feature selection and optimize neural network parameters
- Highlighted the potential of the fusion approach to support initial and exact analysis of AD, aiding clinical decision-making

The structure of this investigation is systematically organized for clarity and coherence. Section 1 covers the overview of research in the introduction section. A comprehensive examination of the literature is given in Section 2, with an emphasis on prior investigations and research gaps. In Section 3, the suggested methodology is described in depth, including the methods and processes used. Results and implications are discussed in Section 4. A description of the main conclusions and contributions is provided in Section 5.

## 2 Related works

AD is a neurological condition that impairs cognition and causes memory deterioration. To detect initial AD, plaques and tau proteins are essential for early diagnosis (11). Using depth-wise separable convolution blocks, mixed skip connections, and sharing weight convolution blocks, this middle-fusion multimodal approach is used for early AD diagnosis. A unique region-of-interest extraction technique for impacted regions and the complete ADNI series is used to assess the system. There are no effective treatments for AD, and patients frequently forget the information that they have learned (12). Neuroimaging and DL algorithms learned on multimodal images were combined in computer-assisted diagnosis (CAD). By concentrating on the gray matter of the brain, an image-based multimodal combination technique was created that increases the accuracy of diagnoses. The method outperforms innovative techniques for AD detection. AD is a neurological disorder that results in cognitive impairment, memory loss, and brain atrophy (13). MRI neuroimaging and a 3D CNN were used in a multimodal image fusion technique to forecast the course of AD. Combining high-dimensional MRI features from accessible sources allows the approach to predict AD progression with an accuracy range of 88.7%–99%. When applied to voxel characteristics derived from MRI, the method performs better than baseline techniques. The proper identification of AD can be difficult, even though it represents a serious health risk (14). Early AD can be detected with the use of multimodal neuroimaging input, such as MRI and PET. However, based on the heterogeneity, these data need appropriate properties. A multimodal fusion-based method analyzes data using transfer learning and the inverse discrete wavelet transform (IDWT). The simulation achieved an accuracy of 81.25% for MRI data and 93.75% for PET data.

They suggested a machine learning (ML) framework for classifying AD patients by combining MRI and PET modalities (15). The approach extracts complementary data and insights by utilizing Same-Subject-Modalities-Interactions (SSMI). The greatest characteristics were employed for categorization, and the SSMI relation is produced from MRI and PET. The outcomes demonstrate unique methods and strong performance metrics, approaching those of single-modality classification tasks. The region analyzed showed a strong correlation with established AD biomarkers. To increase the precision of diagnosing AD and subjective memory complaints (SMC), a portable network was identified (16). To locate discriminative brain regions, the multiscale long-range receptive field is adaptively recalibrated. To identify AD and SMC, it separates the position as well as the magnitude of correlations between 18-FDG-PET and MRI. Making use of FDG-PET and MRI, the approach is the DL investigation technique for diagnosing SMC with FDG-PET, achieving 97.67% accuracy in AD diagnosis assessments. A thorough method for detecting AD, a neurodegenerative brain disease, covers feature extraction procedures, fusion techniques, scalability, and ML approaches for creating an ML-based AD diagnosis system (17). Additionally, ML workflows were compared using thematic analysis to find possible diagnostic solutions. The development of early diagnosis technologies is advanced by the investigation. Multimodal brain imaging is used to diagnose AD utilizing DL algorithms. The findings demonstrate the ability of CNN and transformer-based models to evaluate multimodal neuroimaging data and identify patterns and characteristics linked to AD (18). Several imaging modalities together increase diagnostic precision and forecast the course of a disease. Despite obstacles such as data heterogeneity, small sample sizes, and low generalizability, the neurodiagnostics for AD continue to be a potential research area. The research uses DL architecture with a combination of MRI and PET scans to propose an approach to diagnosis for AD (19). For categorization, the framework employs an Ensemble Deep Random Vector Functional Link (RVFL) and a 10-layer CNN. The metrics perform better than single-layer feedforward networks and conventional classification systems.

The Dual-3DM^3^-AD model was developed to enhance early Alzheimer's detection through MRI and PET data fusion (20). It utilized advanced preprocessing, segmentation, and feature aggregation modules for effective analysis. The model achieved 98% accuracy, 97.8% sensitivity, 97.5% specificity, and 98.2% f-measure, outperforming conventional methods. However, it was constrained by limited dataset diversity and high computational intensity.

The proposed model automated glioblastoma segmentation from MRI scans using a multi-view 2D U-Net with conditional random field (CRF) refinement (21). Axial, sagittal, and coronal slice predictions were combined to capture inter-slice information effectively. High Dice scores of 0.77, 0.90, and 0.84 were obtained for ET, WT, and TC, respectively. Some limitations were observed in the generalization capability and computational efficiency.

A DL framework for glioma segmentation and survival prediction using MRI data. A 2D CNN with a majority rule approach was applied to enhance segmentation reliability (22). Radiomic features were analyzed using a 3D replicator neural network to predict patient outcomes. The model achieved high accuracy on the BRATS2020 dataset but was limited by dataset diversity and clinical data omission.

The performance of different CNN models on brain MRI classification for brain tumor and Alzheimer's disease was evaluated based on data complexity (23). Four CNN models (S-CNN, ResNet50, InceptionV3, and Xception) were implemented using stratified five-fold cross-validation with and without principal component analysis (PCA) after preprocessing the data for class balancing and complexity estimation. The results showed that ResNet50 achieved the highest accuracy of 94.3% and F1-score of 0.93, while S-CNN performed the lowest with 87.5% accuracy, and the evaluation was limited by dataset size, heterogeneity, and the exclusion of other CNN variants.

A mobile phone rating classification system to assist consumers in making informed purchasing decisions (24). A novel dataset of over 13,000 reviews was created from Flipkart, and a federated deep neural network (FDNN) using TF-IDF features was employed, involving two clients and one server across three experimental rounds. The approach achieved an accuracy of 96.68% on the aggregated server while preserving customer data privacy. However, the study was limited to Flipkart reviews, included only two clients, and requires further validation on other e-commerce platforms. An Improved Glowworm Swarm Optimization (IGWSO) was proposed to enhance Parkinson's disease prediction using radial basis function networks (25). The approach optimizes network parameters for improved classification, achieving a high accuracy of 95.3%. While effective, the method is limited to Parkinson's disease, and its applicability to other neurodegenerative disorders remains unexplored. Research investigated early Alzheimer's disease detection using the MssNet model with spatial attention to highlight disease-relevant brain regions (26). The model attained 95.6% accuracy and 94.8% F1-score, outperforming standard CNNs. The findings confirmed the benefit of attention mechanisms in AD diagnosis. However, the research was limited to the MRI modality and lacked multimodal validation.

### 2.1 Research gap

While multimodal fusion techniques using MRI and PET have seen substantial improvement in detecting Alzheimer's disease (15–20), the majority of the current models continue to struggle with challenges related to data heterogeneity, limited sample diversity, and generalization across populations. Additional limitations of current deep learning systems include the lack of adaptive feature selection, the lack of cross-modal interaction modeling, and the limits of precision diagnosis and prediction of neurodegenerative disease (17, 18). Moreover, while high accuracies have been reported on an individual basis (20), the robustness of systems under real-life clinical settings has not been addressed. Therefore, a need has emerged for a generalizable and computationally efficient multimodal framework capable of diagnosing AD early and predicting progression consistently (14, 19). The proposed GWS-SMAtt-ECNN approach solves all the problems by embedding GWS, SMAtt, and ECNN to improve feature extraction, address the heterogeneity of multimodal data, and enhance interpretability, ultimately making it possible to combine MRI and PET imaging techniques to accurately diagnose AD early.

## 3 Methodology

**Figure 1:**
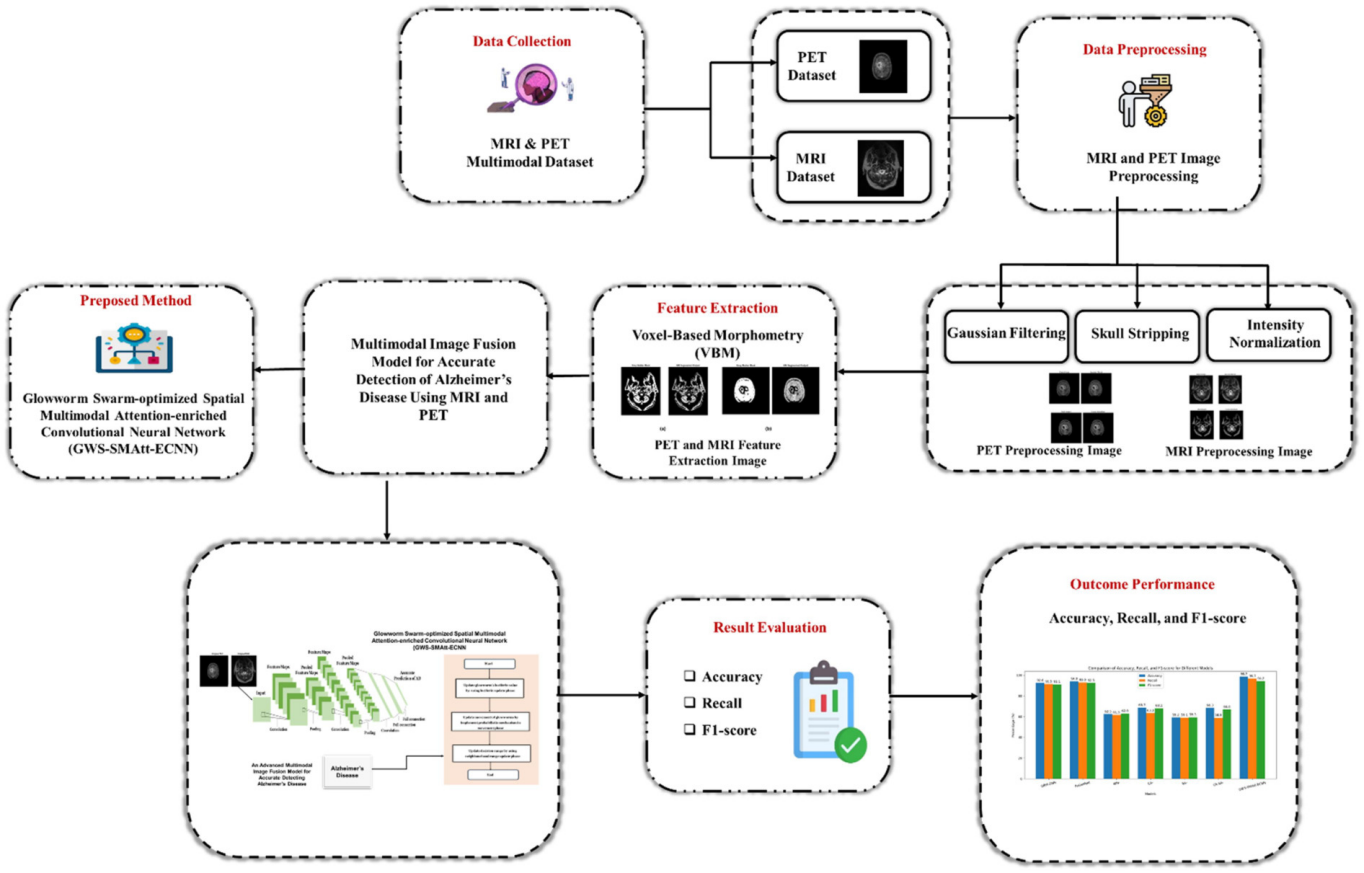
Workflow of the proposed multimodal AD detection framework.

The methodology integrates preprocessing (Gaussian filtering, skull stripping, and intensity normalization), VBM-based feature extraction to describe multimodal image fusion, and a GWS-SMAtt-ECNN model that fuses MRI and PET data using SMAtt and GWS for robust, accurate AD classification. The process of the suggested multimodal ad identification system is shown in Figure 1.

### 3.1 Data gathering

**Figure 2:**
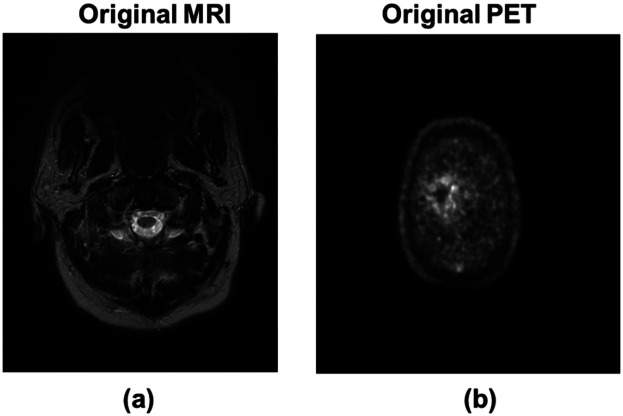
Example of **(a)** MRI and **(b)** PET data images from Kaggle.

The MRI and PET multimodal dataset was gathered from the open-source Kaggle website (https://www.kaggle.com). The dataset comprises structural magnetic resonance imaging (MRI) and functional 18-fluorodeoxyglucose positron emission tomography (FDG-PET) scans obtained from the Alzheimer's Disease Neuroimaging Initiative (ADNI), encompassing images of healthy controls, patients with mild cognitive impairment (MCI), and individuals diagnosed with AD. Each category contained a balanced number of samples (AD, 300; MCI, 300; SMC, 300; NC, 300), ensuring that the training and validation subsets were not biased toward any single class. This subset contains 1,200 multimodal image pairs (MRI and FDG-PET) collected from 300 unique participants distributed across three diagnostic groups: cognitively normal (CN) = 100, MCI = 100, and AD = 100. Each participant contributed multiple scan sessions to represent longitudinal variation, allowing both intra-subject and inter-class learning during model training. Figure 2 shows the sample data images in the dataset.

### 3.2 Data preprocessing

Preprocessing enhances MRI–PET image quality using Gaussian filtering for noise reduction, skull stripping to isolate brain regions, and *Z*-score normalization to standardize intensities, enabling robust feature extraction and accurate AD.

#### 3.2.1 Gaussian filtering

Image preprocessing can improve image quality and feature generation, and it is a crucial step in the accurate diagnosis of AD in MRI scans. The Gaussian filter is an effective preprocessing technique for reducing noise and smoothing MRI images, providing a single illustration. The Gaussian filter, which is used to identify noise in MRI images, is superior at reducing image intensity fluctuations. This filter is less effective at removing salt-and-pepper noise. The filter is based on a Gaussian distribution, leading to a weighted average of the pixel values that tends to prefer the center pixel. The probability density function of the Gaussian distribution, used to compute the filter kernel, is represented in Equation 1:

$$O(w)=\frac{1}{\sqrt{2\pi \sigma^{2}}}f^{-{\left(w-\mu \right)}^{2}/{\left(2\sigma \right)}^{2}}$$ (1)

*w* represents the grayscale intensity of the MRI image, *μ* is the mean intensity value, and *σ* is the standard deviation, which determines the degree of smoothing.

#### 3.2.2 Skull stripping

Skull stripping is a preprocessing technique used in MRI analysis that involves eliminating non-brain tissues, such as the eyes, scalp, and skull, to obtain brain images. By removing only the brain, skull stripping improves the accuracy of AD detection, making it a crucial preprocessing step in every investigation and Diagnostic and Statistical Manual of Mental Disorders (DSM) usage, enhancing feature extraction, reducing image noise, and improving image fusion and classification algorithms.

#### 3.2.3 *Z*-score normalization

The multimodal imaging data (MRI and PET) used in detecting AD are first processed with intensity normalization, specifically using *Z*-score normalization. This method removes the mean and scales the features to unit variance, allowing MRI and PET outputs, often on different scales, to equally inform the diagnosis. Outliers in the imaging data can significantly affect the mean and standard deviation estimates, potentially hiding true pathological variation. As a result, using *Z*-score normalization can reduce the impact of distortion and produce a more robust and sensitive model, particularly regarding subtle anatomical and functional changes, using Equation 2:

$$\hat{A}=\frac{T-F(A)}{Std(A)}$$ (2)

The audio feature *A* is characterized by computing its mean F(A) and standard deviation Std(A).

**Figure 3:**
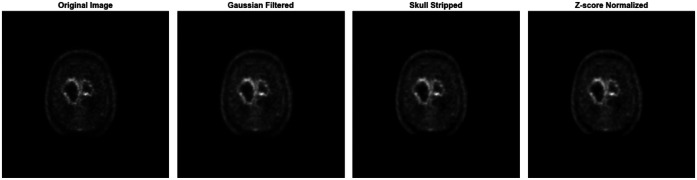
Preprocessing images of PET.

In detecting AD using PET images, preprocessing procedures such as Gaussian filtering, skull stripping, and intensity normalization improve image quality by enhancing image clarity, removing non-brain tissues, and standardizing voxel intensities, leading to accurate analysis with precise feature extraction to provide better disease classification. Figure 3 shows the preprocessed images of PET.

**Figure 4:**
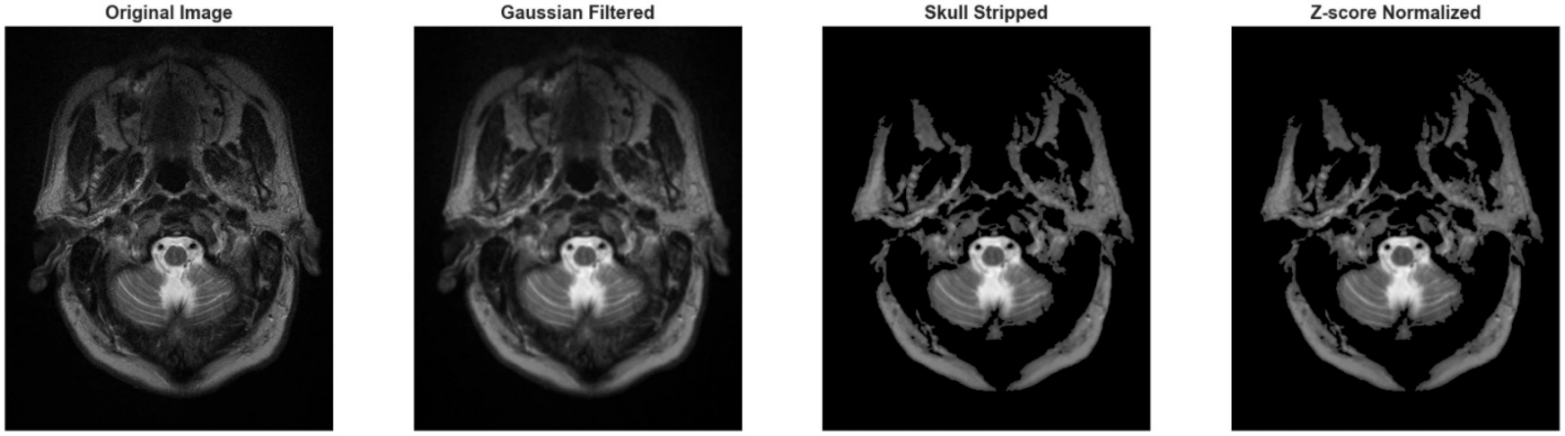
Preprocessed images of MRI.

These preprocessing steps improve image quality, remove non-brain tissue, and normalize intensity values, which increases reproducibility in feature extraction and ultimately improves diagnostic accuracy. Figure 4 shows the preprocessed images of MRI.

### 3.3 Feature extraction using VBM

VBM allows focal differences in brain structure to be assessed and is essential for evaluating neurodegenerative alterations relevant to AD. In the investigation, VBM can analyze MRI data to identify regionally-patterned atrophy and, in particular, the distribution of GM density changes relevant to the progression of AD. The integration of VBM-based gray matter (GM) extraction and PET metabolic mapping was guided by established neurobiological evidence that GM atrophy and glucose hypometabolism are core indicators of Alzheimer's progression. Structural MRI provides high-resolution visualization of atrophic changes in regions such as the hippocampus and temporal cortex, while FDG-PET captures complementary functional deficits in glucose metabolism. By focusing on these biologically relevant features, the model ensures that the fused representation captures both anatomical and functional markers of AD, providing a strong clinical justification for the proposed feature selection and fusion approach.

**Figure 5:**
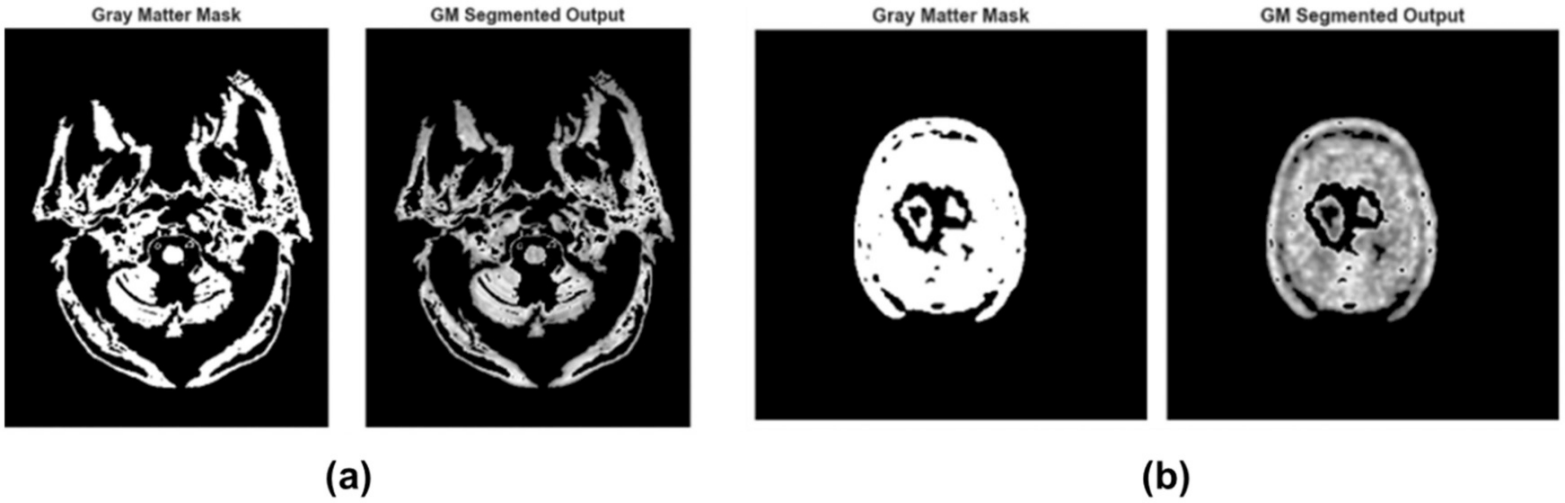
Feature extracted image of **(a)** PET and **(b)** MRI.

The feature extraction images of PET and MRI are shown in Figure 5. The VBM process involves several key steps:

- Spatial normalization: Spatial normalization is an important feature extraction step in VBM for AD detection using MRI and PET scans. It ensures anatomical consistency by transforming brain images to the same stereotactic space. PET scans are mapped to a brain template, co-registered with MRI scans, and normalized for statistical power through group-level comparisons.
- Segmentation: PET scans are employed in the identification of AD, characterized by progressive GM atrophy in the hippocampus and cortex. MRI images are segmented into three categories: GM, cerebrospinal fluid (CSF), and WM. Following segmentation, the images are examined to measure variations in tissue thickness. These images are combined with functional information from the PET scan for a better diagnosis.
- Smoothing: PET and MRI have a critical role in AD detection. The differences in anatomy and smoothing of the segmented GM images were carried out. This maximally reduces the anatomical variability and provides the data with a normal distribution, which is a requirement of parametric statistical testing. PET-based images were used to identify and classify regional atrophy in early stages of AD, providing multimodal information for diagnosis and classification.
- Statistical analysis: Using PET imaging, voxel-wise statistical estimation based on the general linear model (GLM) is employed to detect structural brain alterations associated with AD. A parametric map is produced, which highlights the differences in GM density between AD patients and healthy controls. PET imaging uses structural mapping to consider the features of metabolic activity, which enhances the precision and resilience of AD identification and forecasting. Figures 5a and b illustrate the feature extracted image of PET and MRI, respectively.

GM regions extracted using voxel-based morphometry (VBM) from MRI are known to undergo atrophy in AD, particularly in the hippocampus, temporal lobes, and cortical regions. FDG-PET provides complementary metabolic information, highlighting hypometabolism in these same regions. By combining structural and functional data, the model captures both anatomical degeneration and metabolic deficits, producing a biologically grounded feature set that aligns with well-established AD pathology. Preprocessed MRI scans are analyzed using VBM to extract the GM regions most affected by AD. The GM mask highlights areas such as the hippocampus, entorhinal cortex, and temporal lobes, ensuring only clinically relevant structural regions are considered. The GM mask is applied to spatially co-registered PET scans, isolating voxel-wise metabolic activity within the same clinically significant regions. This ensures that only functionally meaningful PET signals contribute to the final representation.

### 3.4 Multimodal image fusion

The proposed mask-coding fusion strategy integrates structural MRI and functional FDG-PET to generate a clinically meaningful and technically robust representation for AD detection. **Mask-coding fusion:** The MRI and masked PET features are combined using a mask-coding strategy. MRI intensities provide anatomical structure, while PET voxel values are modulated by the GM mask. The fused feature map is created via element-wise multiplication and concatenation to maintain spatial correspondence and integrate complementary information from both modalities.

### 3.5 Proposed technique

The fused multimodal features are input to the GWS-SMAtt-ECNN model. The **SMAtt** module emphasizes brain regions most relevant to AD, enhancing discriminative feature representation. **GWS** selects the most informative features, improving classification accuracy, generalization, and robustness against noise or irrelevant information. The GWS-SMAtt-ECNN model combines GWS for optimal selection of features with SMAtt, which gives attention to brain regions in fused MRI and PET for early AD classification. As an ECNN architecture, the model enhances early AD classification by using robust spatial features and adaptively selecting the most informative subspace while improving overall classification accuracy and model generalizability in the classification of multi-stage AD.

#### 3.5.1 SMAtt

The SMAtt in AD detection is to intensify the model's attention toward important brain regions found in MRI and PET images that are most relevant for identifying neurodegeneration. The focused attention helps identify discriminative spatial patterns related to AD biomarkers such as hippocampal atrophy and hypometabolism. To impose SMAtt in a restricted manner, parameters are required, two efficient and parameter-free operations: channel-wise max pooling (CMP) and channel-wise average pooling (CAP). The concatenated multimodal feature map $W_{\mathrm{cat}\;}\in \;R^{G\times X\times 2H}$, derived from fused MRI and PET modalities, is processed through CAP and CMP to generate SMAtt maps $\omega_{\mathrm{avg}},\omega_{\max}\in R^{G\times X\times 1}$. The spatially enhanced feature representation $W_{\mathrm{cat}}^{x}$ is obtained by element-wise multiplication in Equations 3–5:

$$W_{\mathrm{cat}}^{x}\;\;=\;W_{\mathrm{cat}\;}⊗\;CAP(W_{\mathrm{cat}\;})\;⊗\;CMP(W_{\mathrm{cat}\;})$$ (3)

where

$$CAP\;(W)\frac{1}{d}\overset{d-1}{\underset{d=0}{\sum }}W_{\;ji}^{d}$$ (4)

$$CMP\;(W)=\max(w_{\;ji}^{1},\ldots ,\;w_{\;ji}^{c})$$ (5)

where element-wise multiplication is indicated by ⊗. Finally, the following formulation can be used to describe the process that produced the final fusion outcomes, as shown in Equation 6:

$$W_{\mathrm{fused}}=\frac{W_{\mathrm{IR}}^{\omega }+\;W_{\mathrm{RGB}}^{\omega }}{2}$$ (6)

where

$$W_{\mathrm{IR}}^{\omega },\;W_{\mathrm{RGB}}^{\omega }=Spliy\;(W_{\mathrm{cat}}^{\omega })$$ (7)

The size of the input feature maps and the calculated feature maps using Equation 7, $XW_{\mathrm{IR}}^{\omega },\;W_{\mathrm{RGB}}^{\omega }\;\in \;R^{G\times X\times D}\;$, are identical in the particular instance.

#### 3.5.2 ECNN

The standard CNNs are effective at extracting features from a single imaging modality; they have several limitations in the context of Alzheimer's disease detection. This fused feature set is then processed through enhanced fully connected layers with dropout and batch normalization, improving the network's ability to detect subtle anatomical and metabolic changes, thereby enabling more accurate early diagnosis of Alzheimer's disease and its stages.

$$\hat{y=softmax\;(W.f_{\mathrm{conv}}(X)+b)}$$ (8)

Equation 8 shows that the standard CNN processes an input image *X* (e.g., MRI or PET) by extracting features through convolutional and pooling layers, denoted as $f_{\mathrm{conv}}(X)$, which are then passed through fully connected layers with weights *W* and biases *b*, and finally transformed by a softmax function to produce the predicted class probabilities $\hat{y}$.

**Figure 6:**
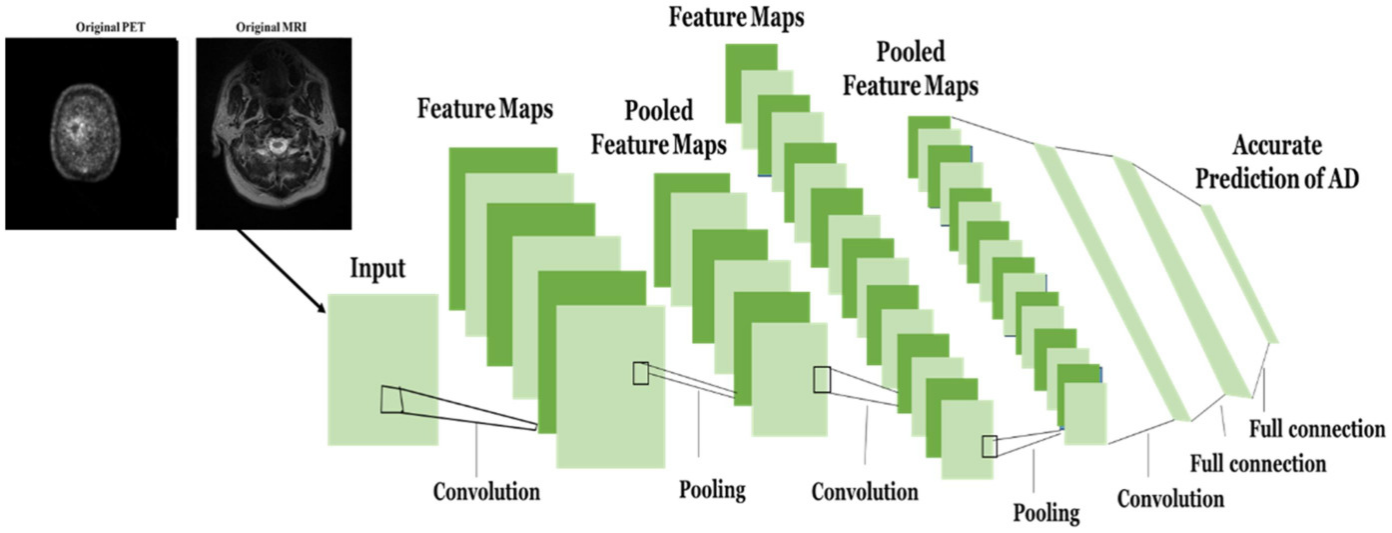
Structure flow of ECNN.

CNNs often struggle to capture subtle changes in brain structure and metabolism, cannot naturally integrate complementary information from multiple modalities, and treat all spatial regions equally, which may reduce sensitivity to disease-relevant areas. The enriched CNN (ECNN) overcomes these limitations by incorporating structural MRI and functional PET scans as multimodal inputs, extracting modality-specific features through parallel convolutional layers. Spatial attention mechanisms are applied to highlight the most relevant regions in each modality, and the resulting features are fused to form a unified representation. The ECNN can identify small changes in the brain and improve its ability to classify by incorporating structural with functional imaging. Compared with the original ECNNs, this approach enables more accurate early diagnosis of AD and its stages. Figure 6 depicts the typical ECNN's construction. The ECNN architecture employed three convolutional layers with filter sizes of 32, 64, and 128, and a kernel dimension of 3 × 3. Each convolutional block was followed by ReLU activation and 2 × 2 max pooling for spatial downsampling. The feature map dimensions evolved as follows: (128 × 128 × 32) → (64 × 64 × 64) → (32 × 32 × 128), where each stage captured progressively abstracted structural and functional information from fused MRI–PET data. Fully connected layers with 256 and 128 neurons were employed for high-level feature integration, followed by a softmax classifier for four-class AD stage prediction. Dropout values between 0.3 and 0.5 were applied to reduce overfitting. Tunable parameters included convolutional filter weights, bias terms, learning rate, and attention weights from the SMAtt module. Additionally, GWS optimization dynamically adjusted the learning rate (0.0001–0.001), luciferin decay rate (0.4–0.6), and neighborhood range (*q*₀ = 1.0 – *q*_t_ = 5.0), ensuring convergence stability.

A variety of convolution filters are used by the ECNN's convolutional layers, frequently referred to as the extraction of features layers, to extract various types of information from the image that was entered. The current layer's convolution filter provides a process of convolution to the input images, resulting in creative feature mappings utilizing the activation function. When using MRI and PET to diagnose AD early, these traits are essential. Equation 9 can be utilized for determining the feature map:

$$w_{i}^{k}=e\left(\underset{\;j\in M_{i}}{\sum }w_{j}^{k-1}*l_{i}^{k}+a_{i}^{k}\right),$$ (9)

where $w_{i}^{k}$ denotes the $i^{th}$ map of characteristics inside the $k^{th}\;$ network layer, $l_{i}^{k}$ denotes the $i^{th}$ multilayer filter, $M_{i}$ incorporates feature maps that connect to $l_{i}^{k}$ in the k−1 layer, $a_{i}^{k}$ is a result of that $i^{th}$ feature map in the $k^{th}$ layer, ∗ symbolizes the 2D convolution procedure, and *f*(·) is the trait that becomes active. ECNN can effectively recover intricate spatial structures from MRI and PET data because of these aspects, which are necessary for the early identification of AD.

The collected feature maps are divided into smaller planes by the pooling layers. This improves the network's capacity for generalization by simplifying its structure and making it impervious to scale, translation, and other forms of visual distortion. Equation 10 shows how the pooling layer is expressed:

$$w_{i}^{i+1}=\beta_{i}^{k}.o(w_{i}^{k}),$$ (10)

where $\beta_{i}^{k}$ is the weight and o(⋅) represents the pooling procedure. Extensive pooling techniques include max pooling and mean pooling. Transported to fully linked layers for the diagnosis of AD, which is necessary to comprehend conceptual frameworks and neuronal connections, followed by Equation 11:

$$z_{j}=sigmoid\;\left(\overset{r}{\underset{g=1}{\sum }}x_{g}.y_{g}+a\right),$$ (11)

where the sigmoid(⋅) procedure restricts the result to a specified range (0,1) and $z_{j}$ is the actual result and usually indicates how probable it is that the $j^{th}$ sample belongs to the AD positive group. Here, *r* is the number of neurons present in the layer before its contents, $y_{g}$ represents the result of the $g^{th}$ neuron, $x_{g}$ is the difference in weight across output neurons and $y_{g}$, and *a* is the bias. The network output for situations involving multiple categories is provided by Equation 12:

$$z_{j}=[z_{\;j1},\;z_{\;j2},\ldots .,z_{\;ji},\ldots ,z_{\;jI}{]}^{s}=softmax(X.Y),\;\;\overset{I}{\underset{i=1}{\sum }}z_{\;ji}=1,$$ (12)

The softmax(⋅) is an establishing function that maps every vector component X.Y to the interval (0,1), making certain that the total of all the components corresponds to 1. $z_{j}$ is the network's response vector for the $j^{th}$ sample, where the $j^{th}\;$ component $z_{\;ji}$ indicates the possibility that the $j^{th}$ sample belongs to the class i. Here, *I* is how many categories there are in total, *X* is the weighted matrix used to merge the output and the previous layer, and *Y* represents the preceding layer's output vector. The class that has the greatest likelihood of $z_{j}$ decides the categorization outcome. If the largest value is $z_{\;ji}$, the sample is classified as Class *i*. To differentiate between various stages or types of AD throughout initial detection, this procedure makes robust multi-class prediction possible.

#### 3.5.3 GWS

**Figure 7:**
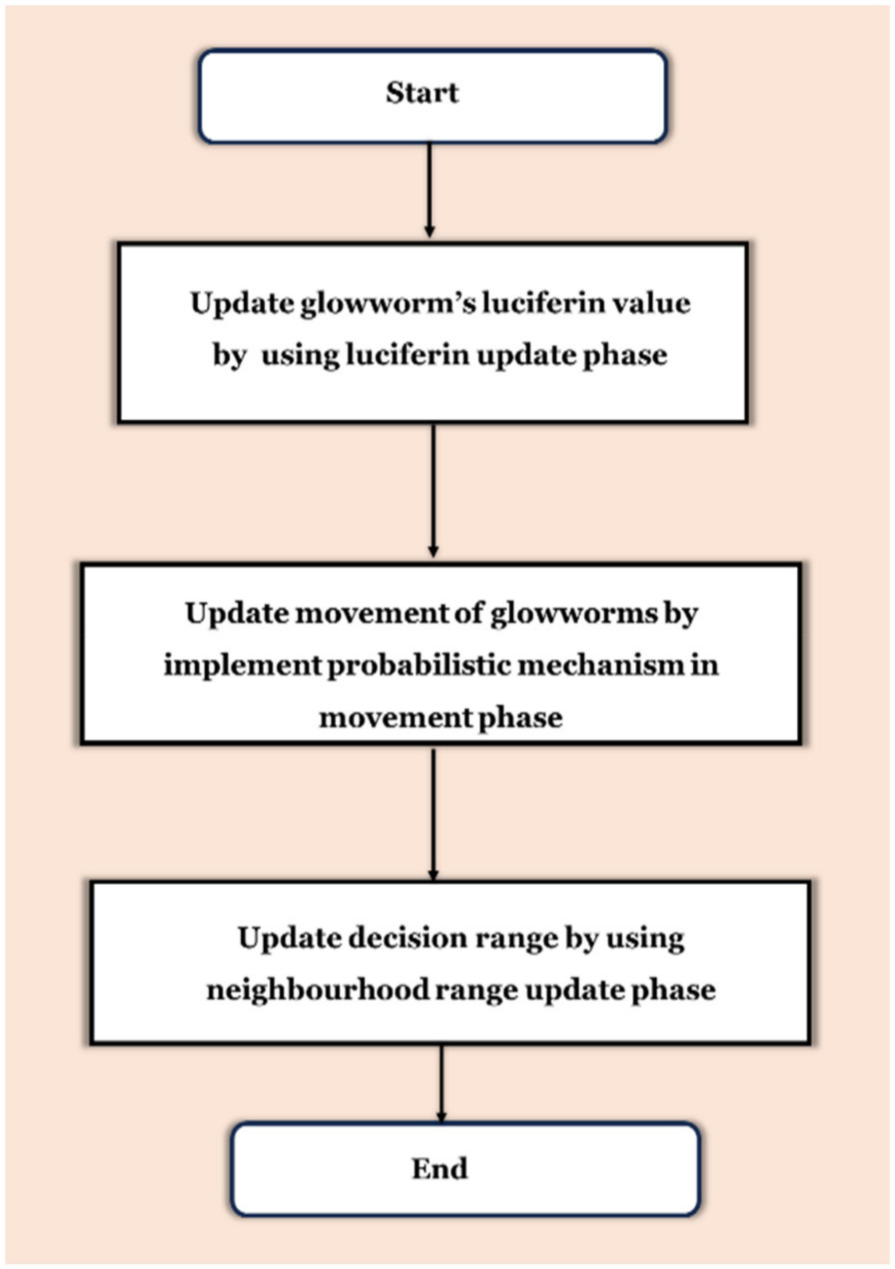
Three main stages of the GWS flow process.

GWS is an algorithm for medical imaging that chooses feature optimization that finds inspiration from biology. GWS supports accurate classification of AD through MRI and PET and aims to improve classification performance by selecting the most pertinent features. GWS by representing the behavior of GW to enhance the exploration quality of solution spaces and improve diagnostic models by optimally selecting feature subsets: the phases of movement, luciferin update, and neighborhood range update. Figure 7 illustrates the GWS flow process's three primary phases.

The luciferin-informed phase is impacted by the function value determined by the glowworm location. To adjust its luciferin level, each GWS adds its initial luciferin value to a quantity proportionate to the fitness in its present position. A portion of the luciferin value is deducted to replicate luciferin decline over time. Equation 13 is used to update a GWS luciferin level:

$$k_{j}\;(s\;+\;1)\;=\;(1\;-\rho )\;k_{j}\;(s)\;+\;zI(w_{j}\;(w\;+1))$$ (13)

where $k_{j}\;(s)$ demonstrates GW's luciferin concentration j at time *s*, ρ is the standard for luciferin degradation (0<ρ<1), *z* is the augmentation of luciferin continuously, and $I(w_{j}\;(w))$ is the goal function's significance when the agent is *j*. By using MRI and PET to diagnose AD early, GWS helps identify more useful feature subsets. That attracts neighbors, indicating feature fitness values containing higher luciferin concentrations. GWS can probabilistically subset the luciferin intensity of neighborly glows as it moves toward them, pausing only upon entirely completing the transition. The process is represented in Equation 14:

$$M_{1}(s)=\{i:c_{\;ji}(s)<q_{c}^{j}(s);k_{i}(s)<k_{j}\}$$ (14)

Equation 13 identifies the GWS neighborhood *j* at time $s.c_{\;ji}(s)$ represents the glowworms' geometric distance from the respective neighbors *j* and *i*, while $q_{c}^{j}(s)$ is the variable neighborhood range for glowworm *j*, constrained with a medical image range such that $0<q_{c}^{j}((s)<q_{t}$. For each GWS *j*, the possibility of approaching a neighbor $l\in M_{j}(s)$ is given by Equation 15:

$$O_{\;ji}=\frac{k_{i}(s)-k_{j}(s)}{\sum_{l\in M_{j}}{\left(s\right)}^{k_{l}}(s)-k_{j}(s)}$$ (15)

The possibility of approaching a neighbor,

$$w_{j}(s+1)=w_{j}(s)+t\left[\frac{w_{i}(s)-w_{j}(s)}{w_{i}(s)-j(s)}\right]$$ (16)

where in Equation 16, t>0 is the step size and ∥⋅∥ denotes the Euclidean norm. $w_{j}(s)\in Q^{n}$ denotes the position of glowworm *j* in an n-dimensional real space at time *s*. The GWS can converge into an area with more luciferin intensity due to flexibility. Initial identification of AD with MRI and PET, this controlled movement enables exploring the feature space efficiently; the swarm could invent the greatest subsets to increase the accuracy of the diagnosis.

Equation 17 controls the adaptive regional range modification, which is necessary for multimodal landscape detection of numerous elevations. Let $q_{0}$ be the initial range, $q_{c}^{j}(s)=q_{0}$. The rule for updates is:

$$q_{c}^{j}(s+1)=min\;\{q_{t},max\;\{\;p,q_{c}^{j}(s)+\beta (m_{s}-|M_{j}(s)|\}\}$$ (17)

This method assists in establishing a balance between exploitation and exploration when choosing features for early AD prediction. The GWS-SMAtt-ECNN models enhance early AD classification by optimizing feature selection, focusing on brain regions in fused MRI and PET, and improving accuracy and model generalizability.

#### 3.5.4 Hybrid improvement strategy

The proposed GWS-SMAtt-ECNN model combines an enriched CNN for extracting multimodal MRI–PET features, spatial multimodal attention to highlight disease-relevant regions, and glowworm swarm optimization to select optimal features. If attention fails, raw features are retained, and local search is applied when no neighbors exist in GWS (Equation 18):

$$F_{\mathrm{fused}}=\;[\;f\;ECNN\;(X_{\mathrm{MRI}})f\;ECNN\;(X_{\mathrm{PET}})]$$ (18)

where $X_{\mathrm{MRI}}\;\mathrm{and}\;X_{\mathrm{PET}}\;$ is the input MRI image data, fECNN denotes the feature extraction function using the enhanced convolutional network (ECNN), and $F_{\mathrm{fused}}$ is the fused multimodal feature map obtained by concatenating ECNN-derived features from both MRI and PET modalities.

$$F_{\mathrm{att}}=\;F_{\mathrm{fused}}⊙(ChannelAvgPool(F_{\mathrm{fused}}))⊙(ChannelMaxPool(F_{\mathrm{fused}}))$$ (19)

**Figure 8:**
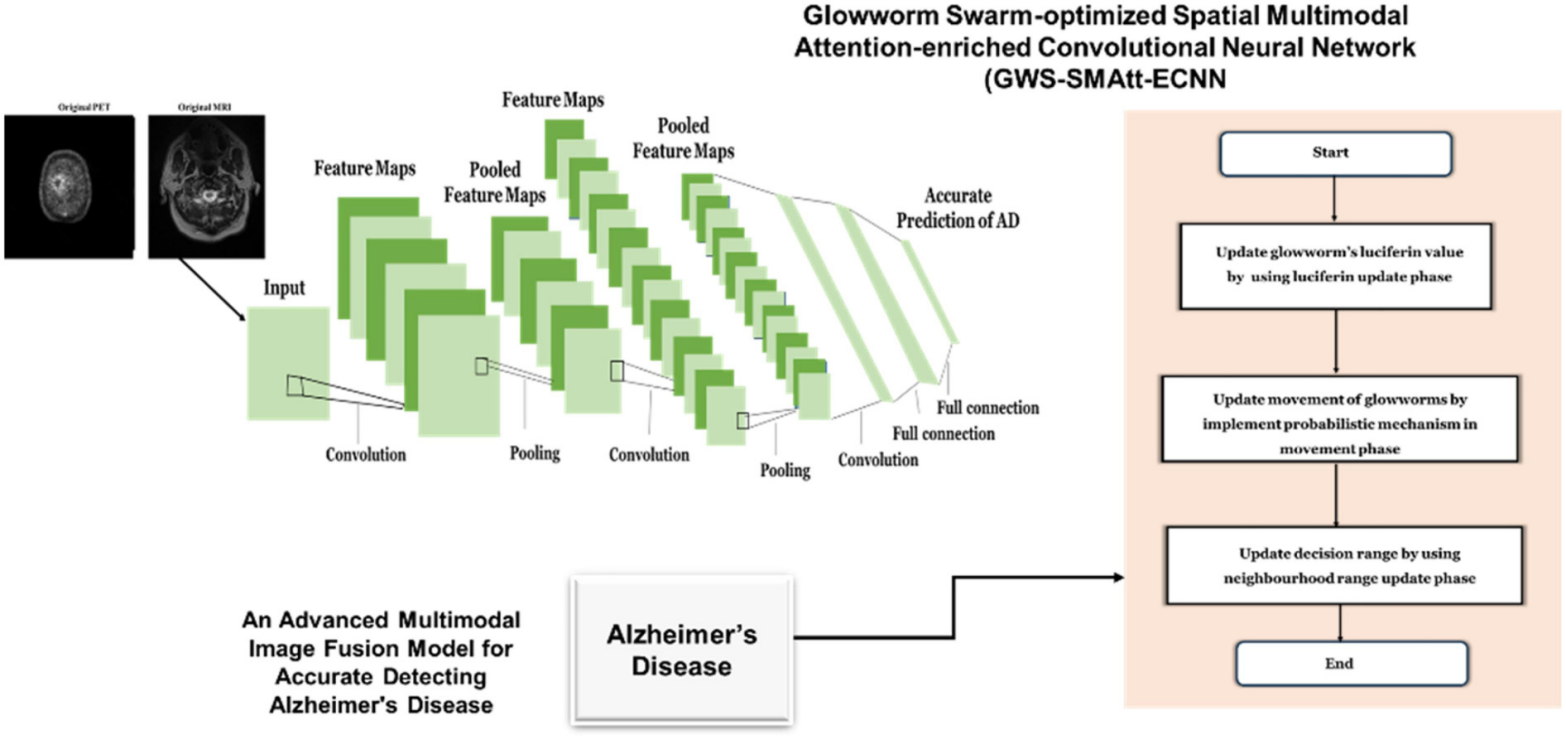
Architecture for the proposed method.

Equation 19 shows where ⊙ is the element-wise multiplication operator, $ChannelAvgPool(F_{\;fused})$ represents the CAP applied to $F_{\mathrm{fused}}$ to capture global average attention weights, $hannelMaxPool(F_{\mathrm{fused}})$ is the CMP applied to $F_{\mathrm{fused}}$ to capture prominent feature activations, and $F_{\mathrm{att}}$ is the attention-refined feature map highlighting the most discriminative features from the $F_{\mathrm{att}}$ representation. Figure 8 depicts that the integrated approach enhances both accuracy and efficiency in Alzheimer's disease classification, enabling precise early detection.

Table 1 summarizes the key hyperparameters used during model training and optimization, and Algorithm 1 shows the hybrid pseudocode for GWS-SMAtt-ECNN.

**Table 1:** Hyperparameters of the proposed GWS-SMAtt-ECNN model.

Hyperparameter	Value/range
Conv filters	32, 64, 128
Kernel size	3 × 3
Activation	ReLU
Pooling	Max/Avg, 2 × 2
Dropout	0.3–0.5
FC layers	256, 128
Attention laps	CAP and CMP
Population size	20–50
Luciferin decay (*ρ*)	0.4–0.6
Step size (*t*)	0.03–0.1
Neighborhood range	*q*₀ = 1.0, *q*_t_ = 5.0
Iterations	100–200
Optimizer	Adam
Learning rate	0.0001–0.001
Batch size	16–32
Epochs	50–100
Loss function	Categorical cross-entropy

Algorithm 1 **Hybrid pseudo code for GWS-SMAtt-ECNN**

InputMRI&PET

Input_MRI,Input_PET=LoadImages()

ECNN

features_MRI=ConvPool(Input_MRI)

features_PET=ConvPool(Input_PET) fused_features=Concatenate(features_MRI,features_PET)

ApplySMAtt

CAP=ChannelAvgPool(fused_features)

CMP=ChannelMaxPool(fused_features)

ifCAPisnotNoneandCMPisnotNone:

attention_map=fused_features⊗CAP⊗CMP Else: attention_map=fused_features#fallbackifattentionfails

ClassifywithECNN

flattened=Flatten(attention_map)

$if\;\;task\;==\;‘‘binary^{\prime \prime }:$

output=Sigmoid(Dense(flattened))

Else:

output=Softmax(Dense(flattened))

OptimizeFeatureSubsetwithGWS

foreachglowworminswarm:

UpdateLuciferin(glowworm)

neighbors=FindBrighterNeighbors(glowworm)

Ifneighbors:

MoveToNeighbor(glowworm,neighbors)

Else:

glowworm.position=RandomSearch(glowworm.position)

UpdateRange(glowworm)

best_features=SelectFeaturesFromSwarm()

TrainFinalClassifier(best_features)

Evaluate()

The GWS-SMAtt-ECNN algorithm fuses MRI and PET features using an enhanced CNN and applies spatial-channel attention to highlight important regions. The fused feature map is flattened and passed to a classifier (sigmoid or softmax based on the task). GWS selects the optimal feature subset by simulating swarm behavior. The final classifier is trained on these optimized features to enhance medical image diagnosis accuracy.

The proposed GWS-SMAtt-ECNN model demonstrates notable improvements in diagnostic accuracy and robustness compared with existing multimodal deep learning approaches. By effectively leveraging complementary structural and functional information from fused MRI and FDG-PET scans, the model provides reliable detection across multiple stages of Alzheimer's disease. The integration of the bioinspired GWS optimization algorithm enhances both feature selection and network parameter tuning. High-dimensional and heterogeneous multimodal imaging data often contain redundant or noisy information that can hinder model performance. GWS simulates the behavior of glowworms to explore the feature space based on luciferin intensity (fitness), enabling the selection of the most informative features while discarding irrelevant ones. Additionally, GWS dynamically adjusts network parameters, improving convergence, generalization, and stability during training. By balancing exploration and exploitation in the feature space, GWS mitigates the challenges of data heterogeneity and high dimensionality, contributing to the superior accuracy, robustness, and reliability of the GWS-SMAtt-ECNN model for early Alzheimer's disease detection.

## 4 Evaluation performance

The results of the model's application are examined in this section, along with comparison analysis and effectiveness assessment (Table 2).

**Table 2:** The hardware and software specifications of the proposed method.

Component	Specification/details
Hardware	NVIDIA GPU (e.g., RTX 3090)
	Intel Core i9 CPU, 32 GB RAM
	1 TB SSD storage
Software	Python 3.10
	PyTorch/TensorFlow
	CUDA toolkit for GPU acceleration
	Libraries: NumPy, SciPy, scikit-learn, OpenCV

### 4.1 Confusion matrix

**Figure 9:**
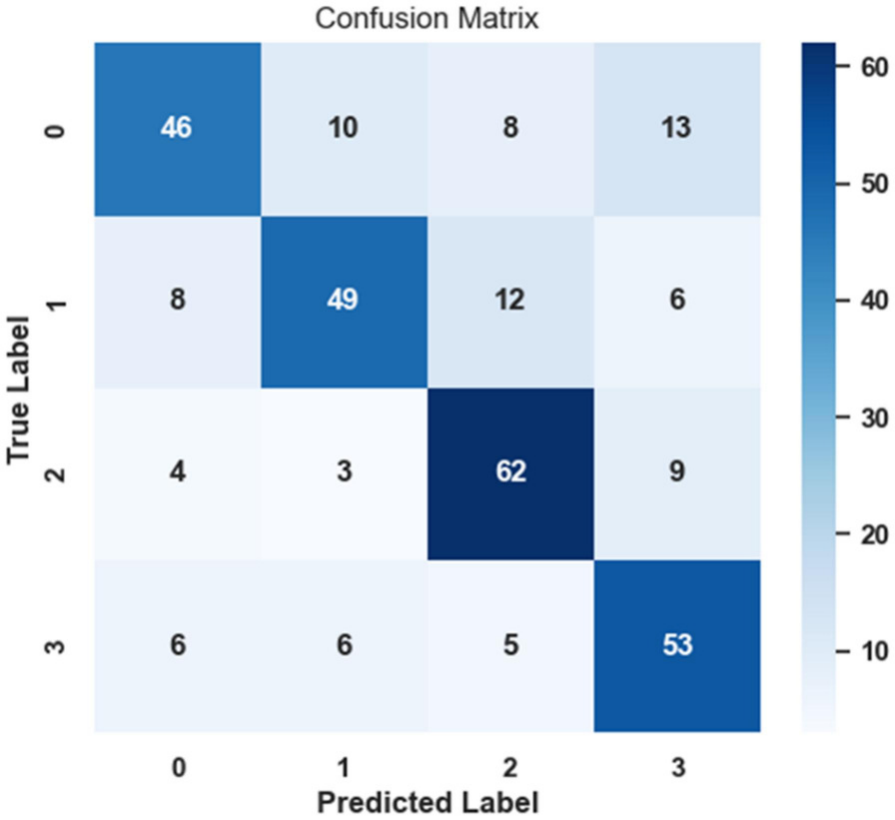
Confusion matrix visualizing classification results using fused modalities.

The suggested GWS-SMAtt-ECNN model's accuracy in classification utilizing combined MRI and PET information is displayed in the confusion matrix, clearly demonstrating the model's ability to classify four AD classes with minimal error. The diagonal dominance indicates reasonable predictive ability in terms of accuracy in all classes. Figure 9 shows the performance of the confusion matrix.

The confusion matrix evaluates the model's classification accuracy by analyzing the predicted and true labels across four classifications. It illustrates errors where the model mistakenly assigns certain labels. The results indicate the model has a good classification rate for the majority of classes, but tends to miss the mark when classifying Class 3. It is concluded that further improvement of the model is warranted to make more accurate predictions, in particular for Class 3.

### 4.2 Accuracy loss

**Figure 10:**
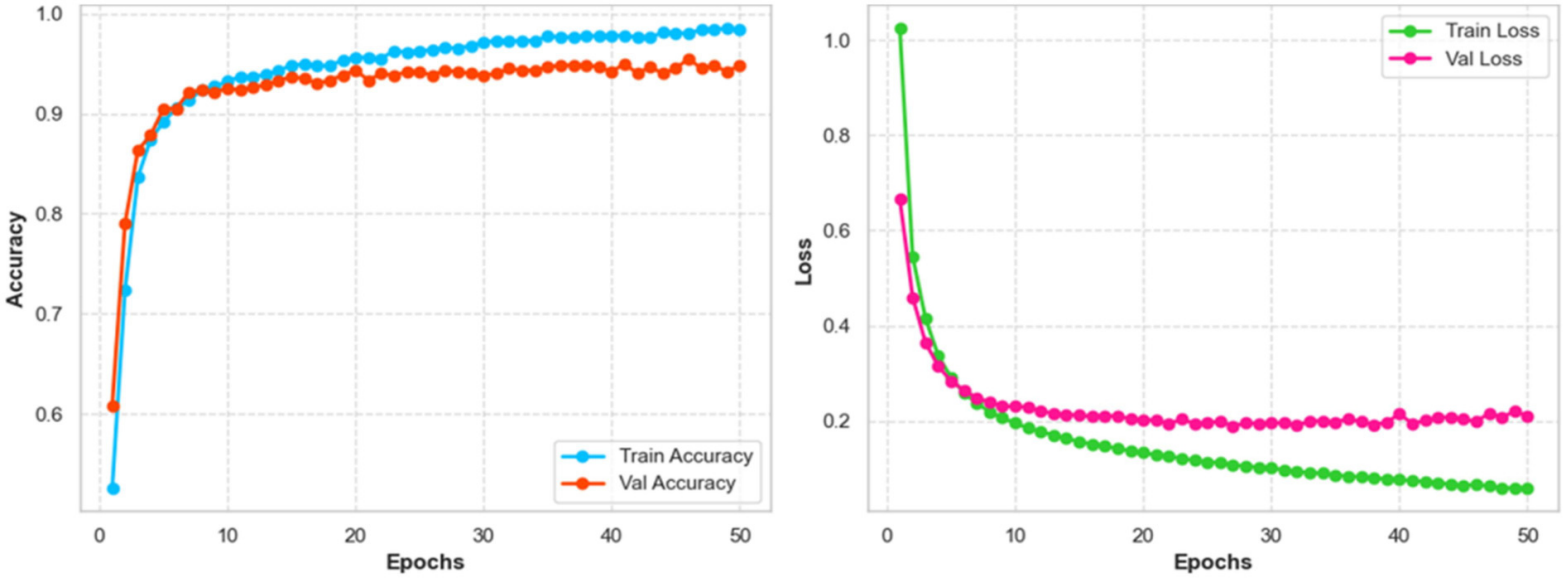
Accuracy and loss trends through training and validation.

The training and validation curves present the results of the GWS-SMAtt-ECNN model performance shown in Figure 10. The training accuracy continued to increase and reached nearly 99% at the end of 50 epochs, while the validation accuracy was sustained well above 94%, promising that the technique performed well. The loss indicated that during retraining in the early epochs, the loss function experienced significant declines, and convergence was achieved with limited overfitting. The results demonstrate that the model is capable of reliably classifying multimodal MRI–PET data to determine accurate and early detection of AD.

### 4.3 Precision-recall curve analysis of AD

**Figure 11:**
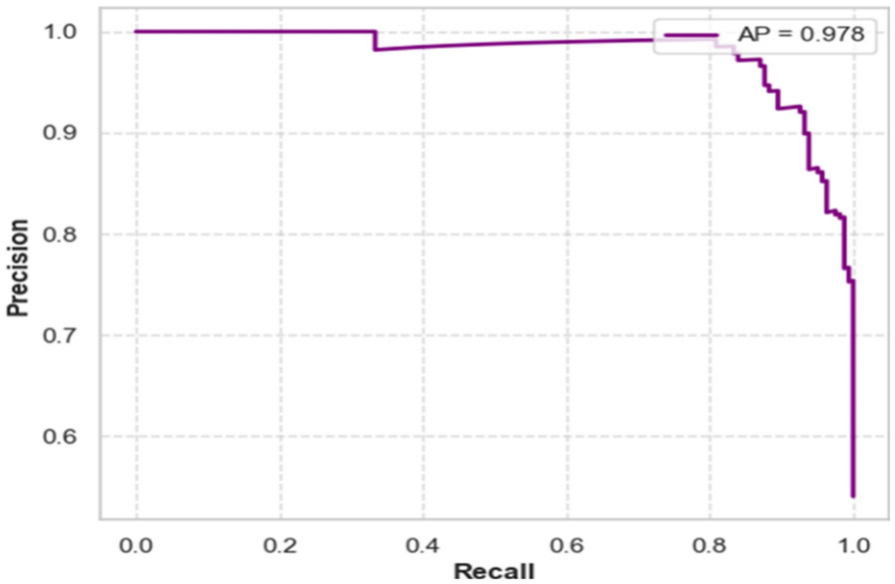
Precision-recall curve demonstrating model performance with high AP.

The precision-recall curve illustrates how well the GWS-SMAtt-ECNN model performed when classifying AD using fused MRI and PET features, as shown in Figure 11. Its capacity to retain a high level of precision at different recall levels is demonstrated by its average precision (AP) score of 0.978. A high precision score indicates limited false positives and demonstrates that the model provides a trustworthy indicator of performance for early diagnosis, which is crucial for appropriate medical attention as AD improves.

### 4.4 Receiver operating characteristic (ROC) curve

**Figure 12:**
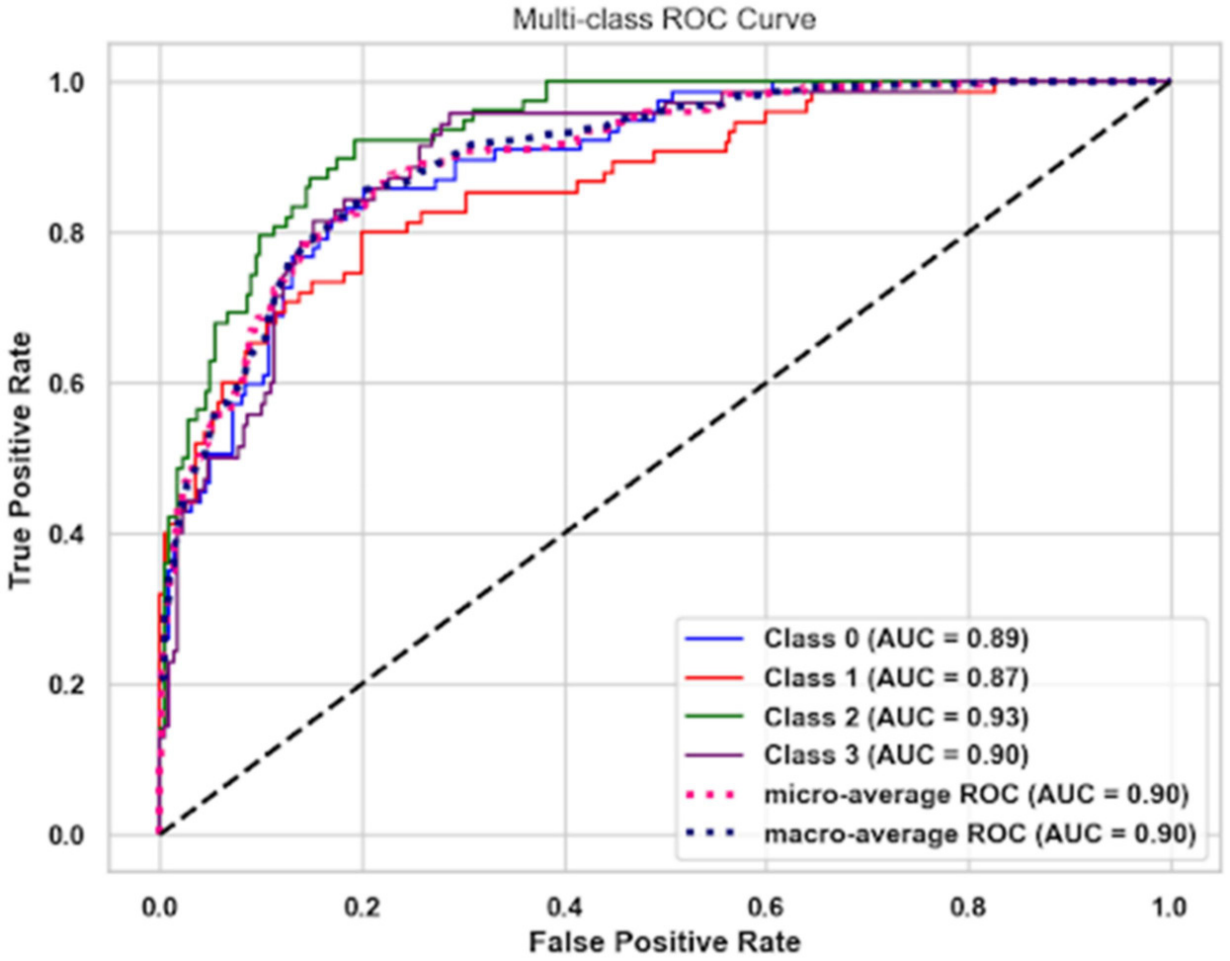
Multi-class ROC curve.

The multi-class ROC curve of the suggested GWS-SMAtt-ECNN model with high discrimination ability for all diagnostic classes. The AUC values ranged from 0.87 to 0.93, with micro- and macro-average AUC scores of 0.90, indicating good overall classification performance (Figure 12).

### 4.5 Per-class performance evaluation

The per-class sensitivity and specificity values are summarized in Table 3. For the fused MRI–PET model, the average sensitivity and specificity across diagnostic categories were 96.1% and 95.9%, respectively, demonstrating balanced and reliable classification performance.

**Table 3:** Per-class sensitivity and specificity of the proposed model.

Diagnostic group	Sensitivity (%)	Specificity (%)
Alzheimer's disease (AD)	97.8	96.9
Mild cognitive impairment (MCI)	95.4	94.1
Subjective memory complaint (SMC)	94.8	95.6
Normal control (NC)	96.5	97.2
Average	**96** **.** **1**	**95** **.** **9**

Bold values indicate the proposed method's reading.

### 4.6 Dataset comparison

Table 4 compares the performance of the proposed multimodal model on two datasets for stroke or brain disorder detection using the real-time stroke detection in MRI, CT, and PET image dataset.

**Table 4:** Performance comparison of proposed and existing datasets for real-time stroke and brain disorder detection.

Metrics	Accuracy (%)	Recall (%)	F1-score (%)
Dataset (real-time stroke detection in MRI, CT, PET images) (proposed)	**98.70**	**96.73**	**94.22**
Dataset (MRI and PET multimodal) (existing)	92.69	90.65	91.20

Bold values indicate the proposed method's reading.

The proposed model achieved superior results with an accuracy of 98.70%, a recall of 96.73%, and an F1-score of 94.22%, demonstrating its effectiveness in identifying true positives and overall classification reliability. In contrast, on the MRI and PET multimodal dataset (existing dataset), the model achieved a lower performance accuracy of 92.69%, a recall of 90.65%, and an F1-score of 91.20%, highlighting that the proposed dataset with multimodal fusion and optimized feature extraction enhances model performance, likely due to richer structural and functional information and improved preprocessing.

### 4.7 Paired *t*-test

Table 5 presents the paired *t*-test results comparing the performance of three models, QS-LGBM, SA-BFRNN, and QuartzNet across key classification metrics.

**Table 5:** Paired *t*-test comparison of QS-LGBM with baseline models on classification performance metrics.

Model	Accuracy (%)	Precision (%)	Recall (%)	F1-score (%)	*p*-value (vs. QS-LGBM)	Significance
QS-LGBM	97.52 ± 0.85	96.80 ± 0.90	97.10 ± 0.88	96.95 ± 0.87	–	–
SA-BFRNN	90.13 ± 1.20	88.50 ± 1.10	89.20 ± 1.15	88.85 ± 1.12	0.002	Significant
Quartz Net	91.45 ± 1.05	90.20 ± 0.98	90.75 ± 1.02	90.48 ± 1.00	0.004	Significant

Both SA-BFRNN and QuartzNet demonstrated comparatively lower accuracy (90.13% and 91.45%, respectively). The *p*-values (0.002 for SA-BFRNN and 0.004 for QuartzNet) were obtained from a paired *t*-test conducted against QS-LGBM, showing statistically significant performance differences (*p* < 0.05). Thus, the improvements achieved by QS-LGBM over the other two models are statistically significant, confirming that its superior results are not due to random variation.

### 4.8 Ablation study

Table 6 shows the performance of different model variants. Incorporating ECNN with SMAtt improves accuracy.

**Table 6:** Performance comparison of model variants on multimodal AD detection.

Model variant	Description	Accuracy (%)
MRI only	ECNN trained using only MRI images (structural data)	91.24
PET only	ECNN trained using only PET images (functional data)	92.67
VBM features only	Model trained solely on VBM-derived gray matter probability maps	89.85
MRI + PET (Simple concatenation)	Direct concatenation of MRI and PET inputs without masking	94.03
Mask-multiplication Fusion (MRI + PET × GM mask)	Hard-coded GM masking applied to PET before fusion	95.78
Mask-soft fusion (*α* = 1)	PET intensity modulated by GM probability map (soft fusion)	96.42
Proposed without SMAtt	GWS + ECNN + mask-coding fusion, but no spatial attention	97.18
Proposed without GWS	ECNN + SMAtt + mask-coding fusion, no feature optimization	97.83
Full proposed GWS-SMAtt-ECNN	Complete model integrating GWS optimization, SMAtt, and mask-coding fusion	98.70

The findings from the ablation studies confirm that each element of VBM-based gray matter masking, SMAtt, and GWS feature optimization offers cumulative contributions toward increased levels of classification accuracy. The full GWS-SMAtt-ECNN model offers an accuracy rate of 98.70%, which is an advantage of 0.87% above the next-best accuracy (which did not include GWS), indicating a combined benefit from utilizing optimization, attention, and anatomically driven fusion.

### 4.9 Comparative evaluation

The comparative assessment shows that GWS-SMAtt-ECNN outperformed the existing models, Gradient Boosting Machine Deep Neural Networks (GBM-DNN) (27) and FusionNet (28), and the models from (29) (NIN, CA, SA, and CA-SA). It achieved the highest performance in comparison with the concerned metrics. Forecasting appeared to be more accurate using GWS-SMAtt-ECNN's superior efficacy in generating fewer false negatives (FN) and false positives (FP). Table 7 presents a comparison between the proposed and existing approaches.

**Table 7:** Comparison between the proposed and existing approaches.

Methods	Accuracy (%)	Sensitivity/recall (%)	F1-score (%)
GBM-DNN (27)	92.6	91.5	91.1
FusionNet (28)	94	93	92.5
NIN (29)	62.50	61.53	62.90
CA (29)	68.70	63.34	68.10
SA (29)	59.17	59.10	59.31
CA-SA (29)	68.33	58.89	66.81
GWS-SMAtt-ECNN (proposed)	**98** **.** **70**	**96** **.** **73**	**94** **.** **22**

Bold values indicate the proposed method's reading.

**Accuracy:** Accuracy measures the overall performance of the model in correctly classifying both AD and non-AD cases. It represents the proportion of total correct predictions out of all cases evaluated. Higher accuracy indicates better general reliability of the model.

**Sensitivity/recall:** Recall, or sensitivity, quantifies the model's ability to correctly identify actual AD patients. It calculates the percentage of true AD cases that are correctly detected. A higher recall ensures fewer positive cases are missed in diagnosis.

**F1-score:** The F1-score balances precision and recall to provide a single performance metric. It is the harmonic mean of precision and recall, reflecting both correctness and completeness of AD detection. A higher F1-score indicates better overall detection performance.

**Figure 13:**
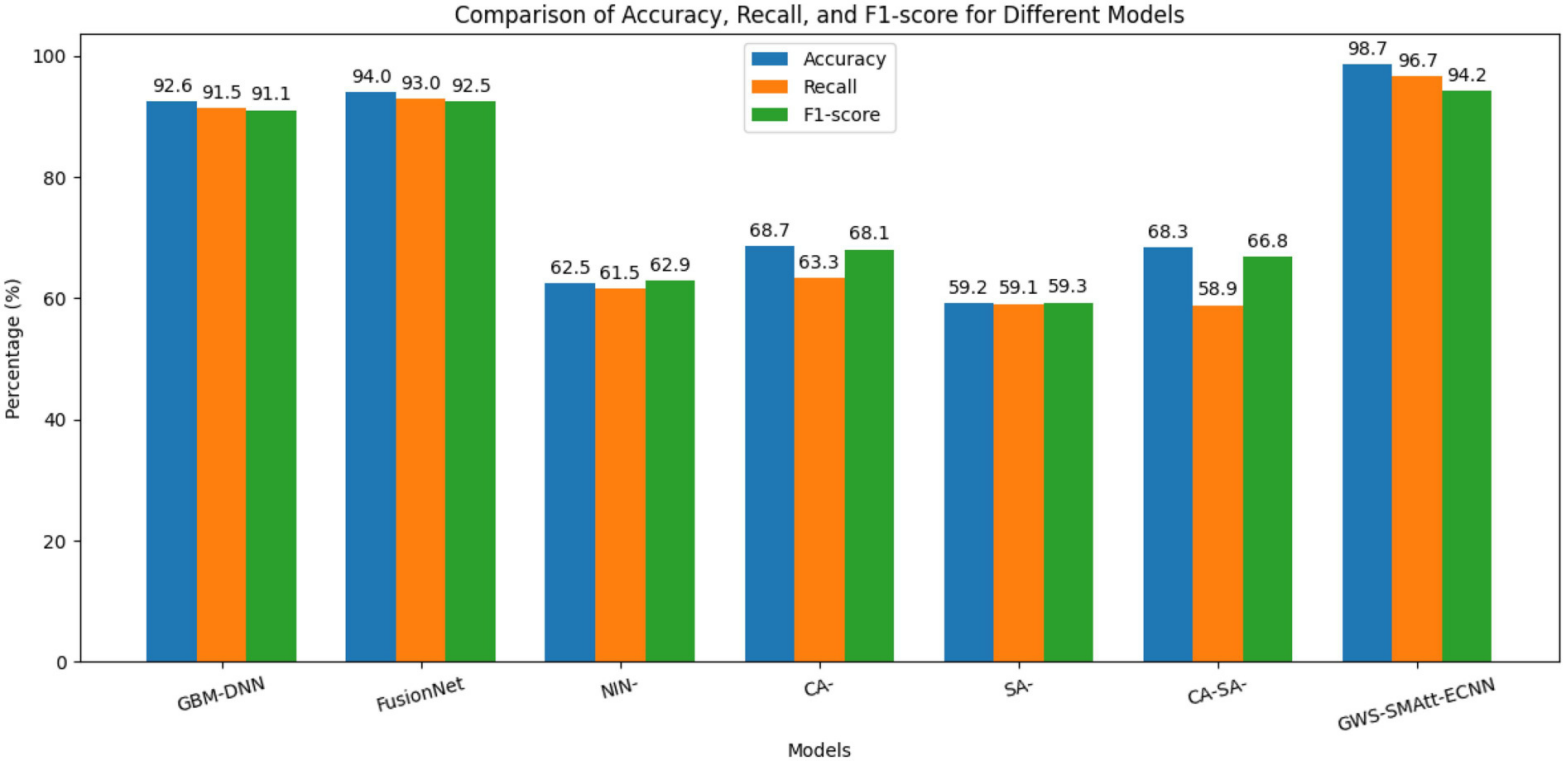
Comparison of accuracy, recall, and F1-score for different models.

Figure 13 presents a comparative performance analysis of various models for Alzheimer's disease detection using multimodal imaging. GBM-DNN (27) achieves an accuracy of 92.6%, a sensitivity (recall) of 91.5%, and an F1-score of 91.1%, while FusionNet (28) improves slightly with 94% accuracy, 93% recall, and 92.5% F1-score. Models from (29) show lower performance: NIN attains 62.50% accuracy, 61.53% recall, and 62.90% F1-score; CA achieves 68.70%, 63.34%, and 68.10%; SA records 59.17%, 59.10%, and 59.31%; and the combined CA-SA achieves 68.33%, 58.89%, and 66.81%, respectively. The proposed GWS-SMAtt-ECNN model significantly outperforms all these methods, reaching 98.70% accuracy, 96.73% recall, and 94.22% F1-score. This demonstrates the effectiveness of the proposed multimodal fusion approach in capturing both anatomical and functional features for accurate AD detection.

### 4.10 Discussion

This research developed a multimodal MRI–PET fusion model using GWS-SMAtt-ECNN to improve Alzheimer's disease detection and classification. CNN model evaluation was constrained by small, heterogeneous brain MRI datasets and a limited number of architectures (23); mobile review classification relied solely on Flipkart data with only two clients, reducing generalizability (24); the IGWSO method was applied only to Parkinson's disease, leaving its effectiveness for other neurodegenerative disorders unknown (25); attention-shift studies involved small sample sizes, limiting applicability to real-world AD patients (26); and some CNN evaluations excluded PCA or multimodal features, restricting performance on complex clinical datasets (23). Previous methods, including NIN, CA, SA, and CA-SA (27), were evaluated for comparison. The NIN model, while effective as a baseline CNN, showed limited performance due to its inability to focus on salient features within the brain images. The CA and SA models, which incorporated channel and spatial attention mechanisms, respectively, improved feature emphasis but remained restricted in handling multimodal data, leading to moderate accuracy and suboptimal generalization. The CA-SA combination provided some improvement by integrating both attention mechanisms, yet it still suffered from modality misalignment and computational inefficiency. Similarly, GBM-DNN (20) demonstrated high computational complexity and overfitting due to its large deep architecture, while FusionNet (21) employed complex fusion layers that increased processing cost and was sensitive to variations in image resolution and quality across modalities. Collectively, these methods faced challenges in generalization, handling limited or imbalanced datasets, and achieving robust multimodal integration. The proposed GWS-SMAtt-ECNN addressed these limitations effectively. GWS enabled efficient selection of the most informative features, reducing computational complexity and mitigating overfitting. The SMAtt mechanism ensured proper alignment between MRI and PET features, minimizing modality misalignment and enhancing feature fusion. Consequently, the model demonstrated higher accuracy, precision, recall, and F1-score compared with all baseline methods. The advantages of the proposed approach included robust generalization to limited and heterogeneous datasets, improved computational efficiency relative to deep architectures such as GBM-DNN, and enhanced discriminative capability for Alzheimer's detection by integrating both structural and functional imaging information. The proposed model is limited by dataset size, modality scope (MRI–PET only), computational demands, attention/optimization variability, and lack of validation on external or real-world clinical datasets.

From a practical perspective, the proposed GWS-SMAtt-ECNN demonstrated the potential to facilitate timely and accurate AD diagnosis in clinical settings. While the model achieved high accuracy, further validation using confusion matrices, AUC-ROC analysis, and statistical testing is recommended for reliability. Its enhanced performance suggests utility in early detection, patient monitoring, and treatment planning. Furthermore, the model's ability to handle imbalanced multimodal datasets indicated its adaptability to diverse clinical populations, supporting its eventual deployment in real-world healthcare environments.

## 5 Conclusion

The combination of MRI and PET scans, a multimodal image integration model that provides sophisticated fusion approaches, improves the diagnostic precision and accuracy of AD diagnosis by providing both structural and functional data on the brain, more efficient feature representation, and greater prospects to identify disease in early stages utilizing DL. The proposed GWS-SMAtt-ECNN technique achieved the highest performance with an accuracy of 98.70%, a recall of 96.73%, and an F1-score of 94.22%. An important drawback to the advanced multimodal image fusing model is that it requires high-quality, co-registered MRI and PET data, which cannot be continuously accessible. Complex models tend to demand a greater computational burden and can be vulnerable to overfitting on small datasets. Future directions involve improving diagnostic accuracy through clinical deployment in real-world settings, adapting to heterogeneous populations, integrating genetic and cognitive biomarkers, and using lightweight models for mobile devices. It can also explore unsupervised learning for early identification of AD in asymptomatic individuals.

## Data Availability

The original contributions presented in the study are included in the article/Supplementary Material, further inquiries can be directed to the corresponding authors.
